# Differences in Prevalence of Transfusion Protocols between Critically Ill Neurologic and Non-Neurologic Patient Populations

**DOI:** 10.3390/jcm12206633

**Published:** 2023-10-20

**Authors:** Thiago M. Oliveira, Michael E. Billington, Raghu R. Seethala, Peter C. Hou, Reza Askari, Imoigele P. Aisiku

**Affiliations:** 1 Department of Emergency Medicine, Brigham and Women’s Hospital, Boston, MA 02115, USA; tmoliveira@bwh.harvard.edu (T.M.O.); mbillington@bwh.harvard.edu (M.E.B.); rseethala@bwh.harvard.edu (R.R.S.); phou@bwh.harvard.edu (P.C.H.); 2 Department of Surgery, Brigham and Women’s Hospital, Boston, MA 02115, USA; raskari@bwh.harvard.edu

**Keywords:** critical care, nervous system diseases, protocols, blood product transfusion

## Abstract

This study describes the prevalence of blood transfusion protocols in ICUs caring for neurologically vs. non-neurologically injured patients across a sample of US ICUs. This prospective, observational multi-center cohort study is a subgroup analysis of the USCIITG—CIOS, comprising 69 ICUs across the US (25 medical, 24 surgical, 20 mixed ICUs). Sixty-four ICUs were in teaching hospitals. A total of 6179 patients were enrolled, with 1266 (20.4%) having central nervous system (CNS) primary diagnoses. We evaluated whether CNS versus non-CNS diagnosis was associated with care in ICUs with restrictive transfusion protocols (RTPs) or massive transfusion protocols (MTPs) and whether CNS versus non-CNS diagnosis was associated with receiving blood products or colloids during the initial 24 h of care. Protocol utilization in CNS vs. non-CNS patients was as follows: RTPs—36.9% vs. 42.9% (*p* < 0.001); MTPs—48.3% vs. 47.4% (*p* = 0.57). Blood product transfusions in the first 24 h of ICU care (comparing CNS vs. non-CNS patients) were as follows: packed red blood cells—4.3% vs. 14.6% (*p* < 0.001); fresh frozen plasma—2.9% vs. 5.1% (*p* < 0.001); colloid blood products—3.2% vs. 9.2% (*p* < 0.001). In this cohort, we found differences in ICU utilization of RTPs, but not MTPs, when comparing where critically ill patients with neurologic versus non-neurologic primary diagnoses received ICU care.

## 1. Introduction

More than 5.7 million patients are admitted to intensive care units (ICUs) in the United States (US) each year, and intracranial hemorrhage or cerebral infarction make up the third most common primary ICU admission diagnosis after respiratory system diagnoses with ventilator support and acute myocardial infarction [1]. Not surprisingly, there has long been an appreciation for the variability of outcomes across different ICU settings. Patient characteristics and ICU volume are two factors contributing to outcome variability [2]. Differing practice patterns between individual providers, specialties that provide critical care, and regional critical care practice patterns may also play a role.

The use of protocols to standardize care delivery in specific clinical scenarios has become prevalent across many ICUs, and some protocols are now required by law [3]. Restrictive transfusion protocols (RTPs) are an example of evidence-based protocols developed following significant research investigations [4,5,6] and increasingly utilized to guide blood product management in ICUs. A restrictive transfusion threshold of hemoglobin <7 g/dL is broadly recognized in critical care for optimizing benefits while reducing potential for harm [5]. While a robust body of research supports RTPs, there continues to be debate surrounding whether the transfusion threshold of hemoglobin <7 g/dL is appropriate for the acutely brain-injured patient and the risks versus the benefits of red blood cell transfusion (RBCT) in acute brain injury [6,7,8,9,10,11,12,13]. More recent evidence supports the use of the restrictive transfusion threshold in critically ill patients with a primary neurologic diagnosis [14,15,16,17].

The applicability of study conclusions informing RTPs for patients with critical neurologic illnesses, such as severe traumatic brain injury, acute ischemic stroke, and subarachnoid hemorrhages, presents a unique challenge. These patients typically represent a small percentage of patients in the development of many protocols and have been excluded or poorly represented in many trials evaluating outcomes after blood product transfusions, including the landmark Transfusion Requirements in Critical Care (TRICC) trial [5]. The aforementioned study, which laid the foundation for restrictive transfusion practices, had a study population wherein only 4.7% of subjects had primary neurologic diagnoses, only enrolled 13% of screened patients into the study, and had no subgroup analysis for patients with brain injury—all limiting the applicability to patients whose primary diagnosis is a neurologic critical illness.

It is unclear whether patients with primary critical neurologic illnesses (herein referred to as the “CNS” group) receive care in ICUs utilizing restricted, protocoled approaches to the transfusion of blood products comparably to patients without primary critical neurologic illnesses (herein referred to as the “non-CNS” group) across ICUs in the United States (US). We posit that the protocols of the specific ICU where a patient is admitted likely affect practice patterns and how that patient is managed. Knowing the practice expectations and practice variations of the ICUs to which CNS patients are admitted offers opportunities for system-based interventions to improve safety, quality-of-care, and patient outcomes. This study compared the presence of established ICU protocols during the care of CNS patients and non-CNS patients; herein, we also explore the association between the presence of ICU blood transfusion protocols and actual blood product transfusion rates.

The US Critical Illness and Injury Trials Group—Critical Illness Outcomes Study (USCIITG—CIOS)—a multi-centered, prospective, observational cohort study—surveyed 69 ICUs across the United States and found a median of 19 protocols for all ICUs and that 93% of the ICUs had 10 or more protocols in place [18]. Leveraging this large multi-disciplinary database, we hypothesized that the prevalence of ICU-based blood transfusion protocols differ when comparing critically ill CNS and non-CNS patients. In this cohort, we found differences in utilization of restrictive transfusion protocols, but not massive transfusion protocols (MTPs), when comparing where critically ill patients with neurologic versus non-neurologic primary diagnoses received ICU care.

## 2. Materials and Methods

Study Setting: The USCIITG—CIOS is a multicenter, prospective observational study of critically ill patients who were admitted to and treated in 1 of 69 participating ICUs in the United States [18]. All participating sites received institutional review board approval for data collection by using a process involving waivers of informed consent [19].

Study Design: This is a retrospective subgroup analysis of the multi-centered prospective observational cohort study USCIITG—CIOS; the data collection methods of the Critical Illness Outcomes Study (CIOS) study have been previously described [18]. Briefly, the CIOS study collected data on several domains including patient demographics, chronic health conditions, diagnosis on ICU admission (limited to primary diagnosis by organ system), acuity of illness on presentation (Acute Physiology And Chronic Health Evaluation II [APACHE II] score), presence of acute organ failure (Sequential Organ Failure Assessment [SOFA] score), acute treatments (including, but not limited to, mechanical ventilation, medications, and blood product transfusions), and outcomes (including disposition). The study also gathered data on participating ICUs’ structure (i.e., medical ICUs vs. surgical ICUs vs. neurologic ICUs) and whether the participating ICUs had specific management protocols in place. ICUs were queried about the presence of protocols for 27 different clinical scenarios, including whether they had a restrictive or massive blood product transfusion protocol in place.

The primary outcome was the presence of restrictive transfusion protocols or massive transfusion protocols. Transfusion thresholds for RTPs and blood product administration guidance for MTPs were determined by each participating ICU and/or hospital. Secondary outcomes included transfusions of packed red blood cells (pRBCs) and fresh frozen plasma (FFP) during the first 24 h and administration of colloids during the first 24 h. This analysis evaluated whether having a primary neurologic vs. non-neurologic diagnosis was associated with an in-place restrictive transfusion protocol and/or massive transfusion protocol. This present study also evaluated whether having a neurologic vs. non-neurologic primary diagnosis on admission to the ICU was associated with blood product or colloid transfusion during the first 24 h of ICU care.

Biostatistical Methods: Descriptive statistics for demographics of CNS vs. non-CNS patients were calculated and then compared using Fisher’s exact Test, chi-squared tests, and t-tests. The CNS and non-CNS groups were treated as dichotomous groups.

## 3. Results

A total of 69 ICUs participated in the study, comprising 25 (36%) medical, 24 (35%) surgical, 20 (29%) mixed-type ICUs. The majority of participating ICUs (64 ICUs; 93%) were located in teaching hospitals. There were 6179 patients across all sites, of which 1266 patients (20.4%) had a primary central nervous system (CNS) diagnosis. The characteristics of 6179 critically ill patients stratified by CNS and non-CNS primary diagnosis enrolled in the Critical Illness and Injury Trials Group—Critical Illness Outcomes Study are presented in Table 1. When stratified by CNS diagnosis, there were differences in the distributions for race, type of ICU, APACHE II scores, and admission sources. The differences in APACHE II scores were not perceived to be significant and were not included in the model. The epidemiologic differences in race also do not have a plausible explainable rationale for the presence of ICU protocols and, therefore, are noted but not included in the model.

**Figure 1:**
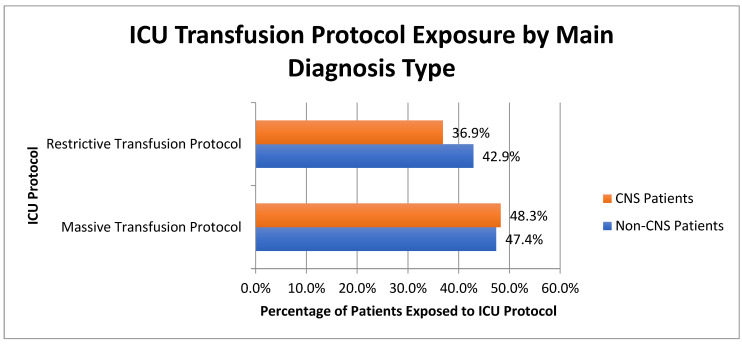
Prevalence of Restrictive and Massive Transfusion Protocols amongst ICU patients with CNS and non-CNS primary diagnoses.

Exposure rates to restrictive transfusion protocols and massive transfusion protocols stratified by neurologic (CNS) versus non-neurologic (non-CNS) primary diagnosis are shown in Figure 1. We found that 467 of 1266 (36.9%) patients with primary CNS diagnoses were admitted to ICUs with restrictive transfusion protocols in place versus 2107 of 4913 (42.9%) patients with all other categories of primary diagnoses (*p* < 0.001), and 611 of 1266 (48.3%) patients with primary CNS diagnoses were admitted to ICUs with massive transfusion protocols versus 2328 of 4913 (47.4%) patients with all other categories of primary diagnoses (*p* = 0.57). Admission to an ICU with RTPs or MTPs in place does not indicate whether patients required any blood product transfusions. Furthermore, compliance with protocols throughout the patient’s ICU stay was not directly measured for each protocol.

**Figure 2:**
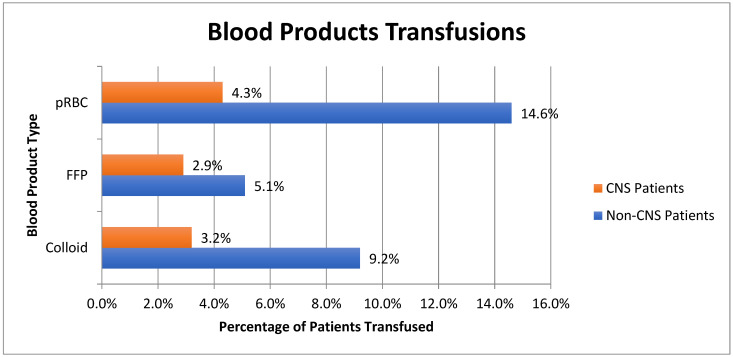
Blood product transfusions amongst ICU patients with CNS and non-CNS primary diagnoses.

These data also show that 54 of 1266 (4.3%) CNS diagnoses received a red blood cell transfusion during the first 24 h of ICU care versus 717 of 4913 (14.6%) non-CNS diagnoses (*p* < 0.001); a total of 36 of 1264 (2.9%) received FFP transfusion compared to 249 of 4890 (5.1%) (*p* < 0.001); a total of 41 of 1264 (3.2%) received colloid blood products compared to 454 of 4890 (9.2%) (*p* < 0.001). Blood product transfusions, categorized by the type of blood product administered and compared across patients with CNS and non-CNS primary diagnoses, are shown in Figure 2.

## 4. Discussion

The majority of ICUs captured in this cohort were in academic centers. This sample had a strong representation of dedicated medical and surgical ICUs with few neurological ICUs—despite 20% of the patient population having primary CNS diagnoses. As the CNS patients were distributed into various ICU types, this study provides novel comparisons of how protocols from general critical care practices are present in the care of patients, with primary neurologic diagnoses being managed in predominantly non-neurologic ICUs. To our knowledge, these comparisons have not been previously described. Protocol utilization is becoming more frequent in numerous clinical settings, though overall prevalence nationwide is variable [18]. The presence of transfusion protocols follows this trend as well.

In our study, we found that a significantly lower proportion of CNS patients were admitted to ICUs with restrictive transfusion protocols compared to non-CNS patients. In contrast, the proportion of CNS and non-CNS patients admitted to ICUs with massive transfusion protocols in place was not statistically different. We suggest the presence of transfusion protocols as surrogates for the practice patterns in participating ICUs within specified clinical scenarios. In this context, these findings suggest two important conclusions. First, CNS and non-CNS patents are exposed to differing practice patterns for routine RBC transfusions in scenarios of anemia without concern for hemorrhagic shock. Second, there is similar practice regardless of principal diagnosis among participating ICUs when there is concern for hemorrhagic shock.

While we found a lower proportion of CNS patients were admitted to ICUs with RTPs compared to non-CNS patients; a different sub-analysis of the USCIITG—CIOS by Seitz, et al. found that older age, white race, post-operative status, hypotension, and acute kidney injury are predictive of admission to an ICU with a RTP [20]. The optimal hemoglobin and transfusion triggers for acute brain injury are controversial [5,6,7,8,9,10,11,12], with the argument that a more liberal approach to CNS patients may have similar systemic complication profiles as non-CNS patients but with organ-specific benefits that outweigh the currently known complications of transfusions in non-hemorrhage scenarios. No studies to date definitively support this hypothesis. To the contrary, recent publications in the literature do not suggest that different protocol approaches should be implemented [13,14,15,16]. Based on these findings, CNS patients should be treated with the same restrictive transfusion threshold as non-CNS patients. Our findings of a difference in exposure to RTPs during CNS patients’ ICU care suggests the importance of education for clinicians in that they should maintain a restrictive transfusion threshold for primarily neurologically critically ill patient even in absence of a formalized protocol. Such educational initiatives are likely especially important given the historical lack of consensus on a transfusion threshold in acute brain injury.

In regard to MTPs, our finding supports the thought that, irrespective of the primary diagnosis, massive hemorrhage requires the preservation of end-organ perfusion and likely the utilization of a massive transfusion protocol in any setting.

Regarding the secondary outcomes, there were statistically significantly fewer transfusions in the CNS group. Despite a smaller proportion of CNS admissions to ICUs utilizing a restrictive transfusion protocol, we found that CNS patients received fewer RBCs, FFP, and colloid transfusions in the first 24 h. The initial 24 h after presentation to an ICU are usually focused on stabilization and resuscitation. The lack of a difference between the two groups with respect to MTPs supports a more uniform approach to ICU patients in shock from acute blood loss. Hemorrhagic shock is not usually a significant component of the initial 24 h ICU course for CNS-injured patients, whereas it is an anticipated component in the course of various patients included in this non-CNS group (i.e., major gastrointestinal hemorrhage or polysystem trauma). Our findings of fewer blood products administered to CNS patients in their early ICU course are consistent with this difference.

A study conducted in 2000 reported that less than 20% of ICUs had transfusion protocols, with no effect on practice from protocol implementation being detected [21]. A decade later, in a sub-analysis of the USCIITG—CIOS, 24 of the 59 ICUs (40.7%) had a RTP and the protocols independently reduced the odds of transfusion in patients with moderate anemia [20]. In broad terms, less than half of ICUs having a RTP speaks to further opportunities for education on the merits of restrictive transfusion thresholds for critically ill patients and to addressing barriers to evidence-based protocol implementation for clinically indicated scenarios. Although a greater percentage of ICUs may have implemented RTPs since then, renewed quantification of RTP prevalence is timely and necessary. Understanding how CNS vs. non-CNS patients are affected by the evolving prevalence and distribution of RTPs is critical as the percentage of patients with neurologic injury is increasing, and advanced stroke therapies have allowed for an increasing number of critically ill patients to survive and require ICU care [22,23,24]. Randomized clinical trials in traumatic brain injury (TBI) and hemorrhagic cerebrovascular accident (CVA) have often included transfusion triggers greater than 7 g/dL. A randomized control trial by Robertson et al. failed to show benefit of erythropoietin or transfusions to maintain hemoglobin concentration greater than 10 g/dL among patients with closed head injury [25]. The study was limited to severe TBI patients from two centers. Although not statistically significant, Robertson et al. did find trends towards both favorable rates of improved neurologic outcome at 6 months and fewer thromboembolic events among patients transfused to maintain a threshold hemoglobin of 7 g/dL versus 10 g/dL. Until further studies identify a mortality or functional outcome, the presence and utilization of transfusion protocols should not differ between CNS and non-CNS patients.

### Limitations

Our secondary analysis of the data has several limitations. The CIOS study has static and longitudinal variables that vary with specific protocols and, therefore, compliance with all protocols throughout patients’ ICU stays was not directly measured for each protocol. Transfusion thresholds for RTPs and blood product administration guidance for MTPs were determined by each participating ICU. Detailed protocol review of each participating ICU for identical thresholds and transfusion guidance was not possible based on data collection. It is possible that significant institutional differences between protocols and biased results for secondary outcomes exist, though this is very unlikely. Although both community and academic medical centers were sampled, the participating institutions were heavily weighted towards academic centers (93%). These analyses cannot account for certain institutional practice patterns surrounding which type of ICU a CNS patient is admitted to, especially if there is no neurosurgical ICU and the ICU is not a mixed-type ICU. Other variables that may influence our observed associations include the size of the hospitals, designation as a trauma center or stroke center, and the capacity of ICUs. Our primary analysis uses chi-squared tests and Fisher’s exact test to evaluate the association between CNS vs. non-CNS primary diagnosis and whether there are RTPs and MTPs in place in the ICUs caring for patients. These statistical methods cannot speak to causality and cannot control for potential confounding. Finally, there could be some component of inconsistent classification for patients with isolated traumatic brain injury (i.e., might be classified as trauma or CNS).

## 5. Conclusions

This study sought to elucidate whether there has been a similar appropriation of the protocoled approach of transfusions of blood products amongst patients with primary critical neurologic illnesses (CNS patients) as compared to patients without primary critical neurologic illnesses (non-CNS patients). We found a significant difference in the proportions of CNS vs. non-CNS patients admitted to ICUs with restrictive transfusion protocols and no difference in the presence of massive transfusion protocols. Future research is needed to understand disease-driven and system-driven differences in the care of patients with neurologic critical illness compared to other ICU populations. Further education on existing protocols in the setting of CNS disease may improve protocol adherence and possibly reduce preventable complications.

## Figures and Tables

**Table 1:** Characteristics of 6179 Critically Ill Patients Enrolled in the Critical Illness and Injury Trials Group—Critical Illness Outcomes Study.

Demographics	CNS Diagnosis	Non-CNS Diagnosis	*p*-Value
	1266 (20.4%)	4913 (79.6%)	
Age (mean)	58 (SD = 18)	60 (SD = 17)	*p* < 0.001
Gender (M)	693 (54.7%)	2760 (52.2%)	*p* = 0.036
Race			*p* < 0.001
Black	323 (25.5%)	1029 (20.9%)	
White	722 (57%)	3458 (70.4%)	
Other	157 (12.4%)	288 (5.9%)	
Not reported	64 (5.1%)	138 (2.8%)	
ICU Classification			*p* < 0.001
Medical	422 (33.3%)	2237 (45.5%)	
Surgical	555 (43.8%)	1624 (33.1%)	
Other	289 (22.8%)	1052 (21.4%)	
APACHE II (mean)	16.2 (SD = 7.3)	16.8 (SD = 7.4)	*p* = 0.047
Admission Source			*p* < 0.01
Emergency Department	724 (57.2%)	2006 (40.8%)	
Hospital	162 (12.8%)	990 (20.2%)	
Operating Room	139 (11.0%)	1092 (22.2%)	
Outside Hospital	210 (16.6%)	607 (12.4%)	
Other	31 (2.4%)	218 (4.4%)	

## Data Availability

Data were obtained from the US Critical Illness and Injury Trials Group (USCIITG) and are available from the authors with the permission of the USCIITG.
